# Remotely-sensed productivity clusters capture global biodiversity patterns

**DOI:** 10.1038/s41598-018-34162-8

**Published:** 2018-11-02

**Authors:** Nicholas C. Coops, Sean P. Kearney, Douglas K. Bolton, Volker C. Radeloff

**Affiliations:** 1 Department of Forest Resource Management, 2424 Main Mall, University of British Columbia, Vancouver, British Columbia V6T 1Z4 Canada; 2 SILVIS Lab, Department of Forest and Wildlife Ecology, University of Wisconsin, Madison, USA

## Abstract

Ecological regionalisations delineate areas of similar environmental conditions, ecological processes, and biotic communities, and provide a basis for systematic conservation planning and management. Most regionalisations are made based on subjective criteria, and can not be readily revised, leading to outstanding questions with respect to how to optimally develop and define them. Advances in remote sensing technology, and big data analysis approaches, provide new opportunities for regionalisations, especially in terms of productivity patterns through both photosynthesis and structural surrogates. Here we show that global terrestrial productivity dynamics can be captured by Dynamics Habitat Indices (DHIs) and we conduct a regionalisation based on the DHIs using a two-stage multivariate clustering approach. Encouragingly, the derived clusters are more homogeneous in terms of species richness of three key taxa, and of canopy height, than a conventional regionalisation. We conclude with discussing the benefits of these remotely derived clusters for biodiversity assessments and conservation. The clusters based on the DHIs explained more variance, and greater within-region homogeneity, compared to conventional regionalisations for species richness of both amphibians and mammals, and were comparable in the case of birds. Structure as defined by global tree height was also better defined by productivity driven clusters than conventional regionalisations. These results suggest that ecological regionalisations based on remotely sensed metrics have clear advantages over conventional regionalisations for certain applications, and they are also more easily updated.

## Introduction

Natural systems are complex and variable over time and space, and understanding the patterns and processes that occur within natural systems, their interactions, and their effects on biodiversity is at the heart of macro-ecology. Scientists’ capacity, however, to ask questions and pose hypotheses about biodiversity across broad spatial scales is limited due to the lack of fine-grained datasets that are systematically produced and consistent over large areas^1,2^. As a proxy for complex environmental variation, scientists and resource managers have developed a variety of ecological regionalisations, which classify a land base into regions characterised by similar environmental conditions^3,4^, ecological processes^5^ and biotic communities^6^. Because conditions of interest are relatively homogenous within regions, regionalisations can provide frameworks for generalization, and stratification, and indicate what is a natural or appropriate management goal for a site^7,8^. These regionalisations then, in turn, provide an underlying basis for systematic conservation planning and environmental management^9^, setting priorities for conservation and protection^10^ and identifying areas which are undergoing unusual perturbations and where management interventions may be required.

When developing a regionalisation, the definition of the clusters and the boundaries that delineate them in time and space is the key challenge, with ongoing debate as to the optimum approach. Issues such as whether the stratifications should be undertaken for specific species or for general-purpose applications, whether the resulting clusters have to be spatially contiguous or can be disjointed, should be nested or non-hierarchical, and if the derived stratification units should subsequently form the basis for management^11–13^ remain unresolved. Historically, the delineation of ecoregions was done by experts integrating a wide range of environmental characteristics, and applying a weight-of-evidence approach^14^. Accordingly, the derived ecoregions are subjective, and often revised and questioned for specific locations^15^. As such, the acceptance of ecoregion maps by resource managers is largely dependent on their information needs and whether a given regionalisation meets their management objectives^16^.

With increases in computing power, and the availability of finer-resolution, spatially-explicit datasets of the environment and its biota^17^, the potential to develop quantitative rather than qualitative regionalisations has increased substantially and such quantitative regionalisations have the benefit that they are more explicit, repeatable, transferable, and defensible than subjective regionalisations based on human expertise^18^. These benefits, in turn, enhance and expand the utility of ecoregions, making them more valuable for certain ecosystem management applications, allowing areas of common environmental characteristics to be grouped, and dissimilar classes compared, as well as supporting quantitative analysis of how unique the delineated regions are, and informing monitoring programs.

**Figure 1:**
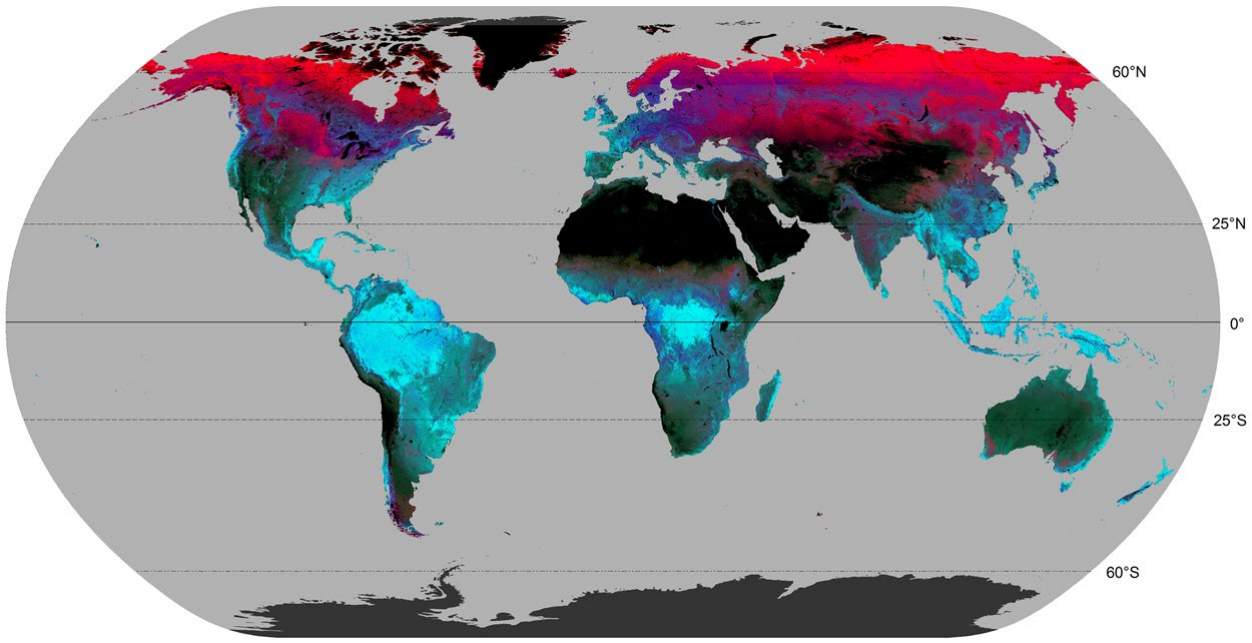
Global Dynamic Habitat Index (DHI) variables shown in colorspace. Red, green and blue colors are assigned to the three DHI variables representing the fraction of photosynthetically active radiation (fPAR) integrated annually, where red is variation of fPAR, green is minimum fPAR and blue is cumulative fPAR. Black regions indicate areas masked from analysis due to insufficient 8-day fPAR observations. Map generated in ArcGIS 10.5 (http://www.esri.com/software/arcgis/arcgis-for-desktop). DHI data is available for download at http://silvis.forest.wisc.edu/data/DHIs and for more information see^51^.

Concurrent with increases in computing resources, advances in remote sensing technology, and long term satellite archives, have increased the relevance of remotely sensed data for ecological studies^19–23^. There are major benefits to the use of remotely sensed data, which include repeatable coverage allowing for consistent and synoptic monitoring, reduced cost (per unit area), and ready access^24,25^. As a result, many macro-ecological studies are now utilizing a range of remotely sensed datasets in survey design, modeling, and regionalisation. For example, Pfeifer *et al*.^2^, in a comprehensive discussion around the use of different remotely sensed products for macro-ecology, highlight the use and misuse of vegetation indices derived from remote sensing observations, such as landscape greenness (computed as the ratio of the red and near infrared regions of the spectrum) and encourages the use of hypothesis development and testing in the use of remote sensing indices. The Dynamics Habitat Indices (DHIs), originally developed by Mackey *et al*.^26^ and Berry *et al*.^27^ in Australia and updated for North America by Coops *et al*.^19^ and globally by Radeloff *et al*.^28^, offer an alternative to simple vegetation indices and provide an opportunity to examine geographic patterns of environmental characteristics (Fig. 1). Specifically, the DHIs capture: (1) cumulative annual productivity as the integrated landscape productive capacity over a year analogous to the available energy hypothesis which suggests that areas of high vegetation productivity have more resources to partition among competing species, thus supporting a greater number of species, and higher population densities, than areas with lower productivity^29–31^ (2) annual minimum productivity as minimum amount of vegetation production over a year, which may compose impositions of inclement climate and seasonally low productivity as constraints on biodiversity and (3) seasonal variation in productivity, which reflects how the vegetation varies within the year, an indicator of climatic variation, and phenology which may be indicative of the capacity of the landscape that may limit permanent resident species^32^, but not migratory species^33^.

Additional insights into environmental characteristics of vegetation condition come from active remote sensing datasets that focus on the vertical structure of vegetation, such as Light Detection and Ranging (LiDAR) technologies. However, LiDAR datasets currently do not provide the same wall-to-wall continental and global coverage offered by optical sensors. Fortunately, the Geoscience Laser Altimeter System (GLAS) aboard the Ice, Cloud and land Elevation Satellite (ICESat) collected waveform LiDAR data from 2003 to 2009 and global wall-to-wall estimates of canopy height have been derived by extrapolating GLAS derived canopy heights through empirical relationships with spatially contiguous variables. For example, Simard *et al*.^34^ used cloud-free GLAS waveform data together with climatic, topographic and other ancillary variables to predict canopy height globally at 1-km resolution.

Here, our goal was to conduct an environmental regionalisation using an unsupervised two-stage multivariate clustering approach based on the newly developed global DHI layers produced from over a decade of consistent, high quality reflectance data produced by the MODIS sensor onboard TERRA and AQUA satellites. Our first objective was to develop a hierarchical clustering of ecologically distinct regions using the DHI layers as primary inputs. Our second objective was to quantify how well these DHI-based clusters can differentiate species richness of three key taxa and global canopy height, in comparison to a conventional regionalisation.

## Results

**Figure 2:**
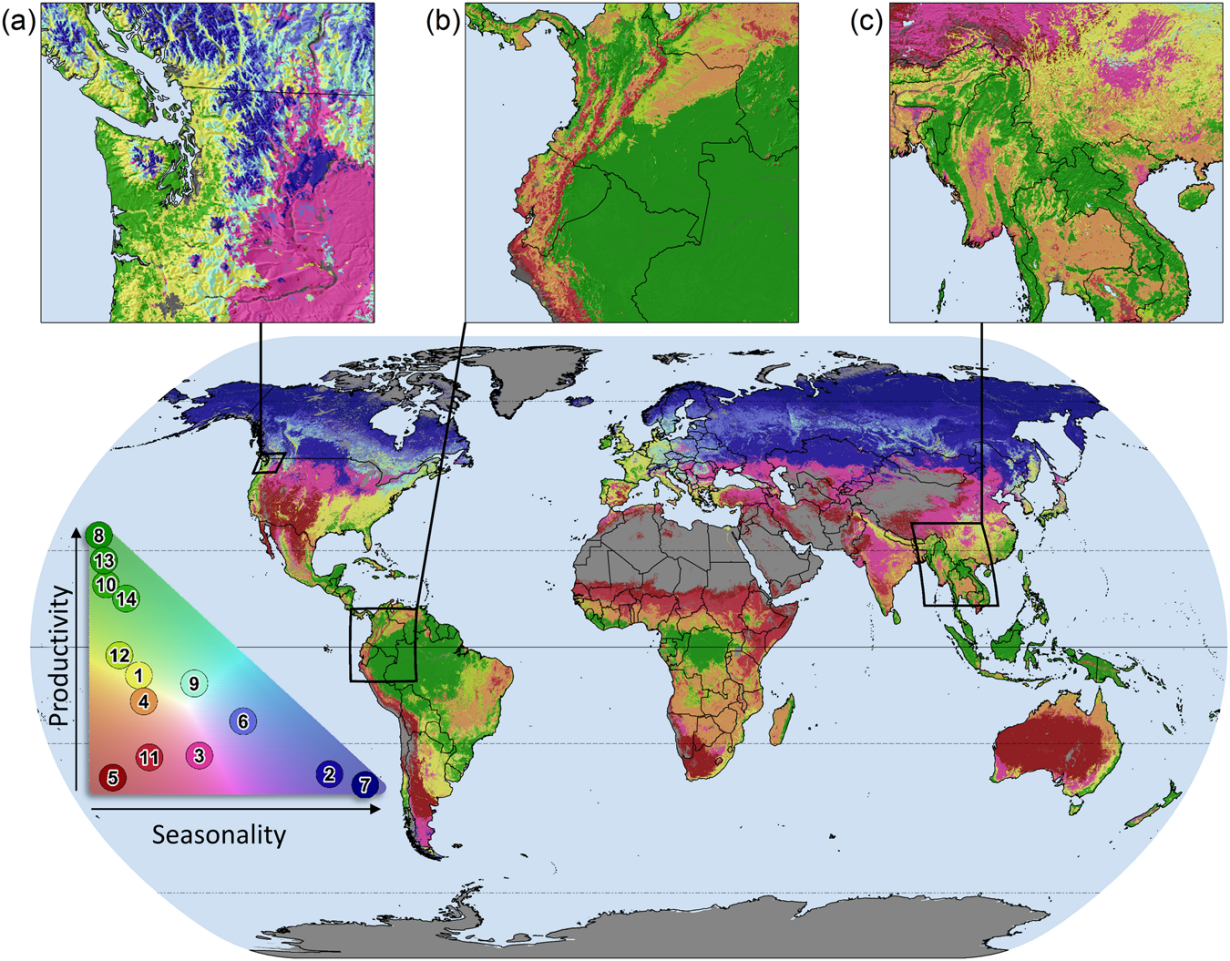
Regionalization map of 14 DHI-derived clusters with examples of fine-scale spatial patterning. Colors were discretely assigned (exactly 14 colors shown) based on each cluster’s mean productivity (cumulative fPAR) and seasonality (variation of fPAR) relative to all other clusters, as shown in the colorspace legend. Green areas indicate higher productivity and lower seasonality (e.g., rainforests), blue areas indicate higher seasonality and lower productivity (e.g., boreal forests), and red areas indicate low productivity and low seasonality (e.g., deserts). Regional productivity patterns corresponding to fine scale drivers (e.g., elevation gradients, microclimates, soil types) are evident in examples from (**a**) the coastal mountains and rain shadow of the Pacific Northwest in North America, (**b**) the Amazon Basin and Andes mountains in South America and (**c**) broadleaf rainforests, coniferous forests and plateaus in Southeast Asia. Map generated in ArcGIS 10.5 (http://www.esri.com/software/arcgis/arcgis-for-desktop). Country boundaries made with Natural Earth. Free vector and raster map data @ naturalearthdata.com.

**Figure 3:**
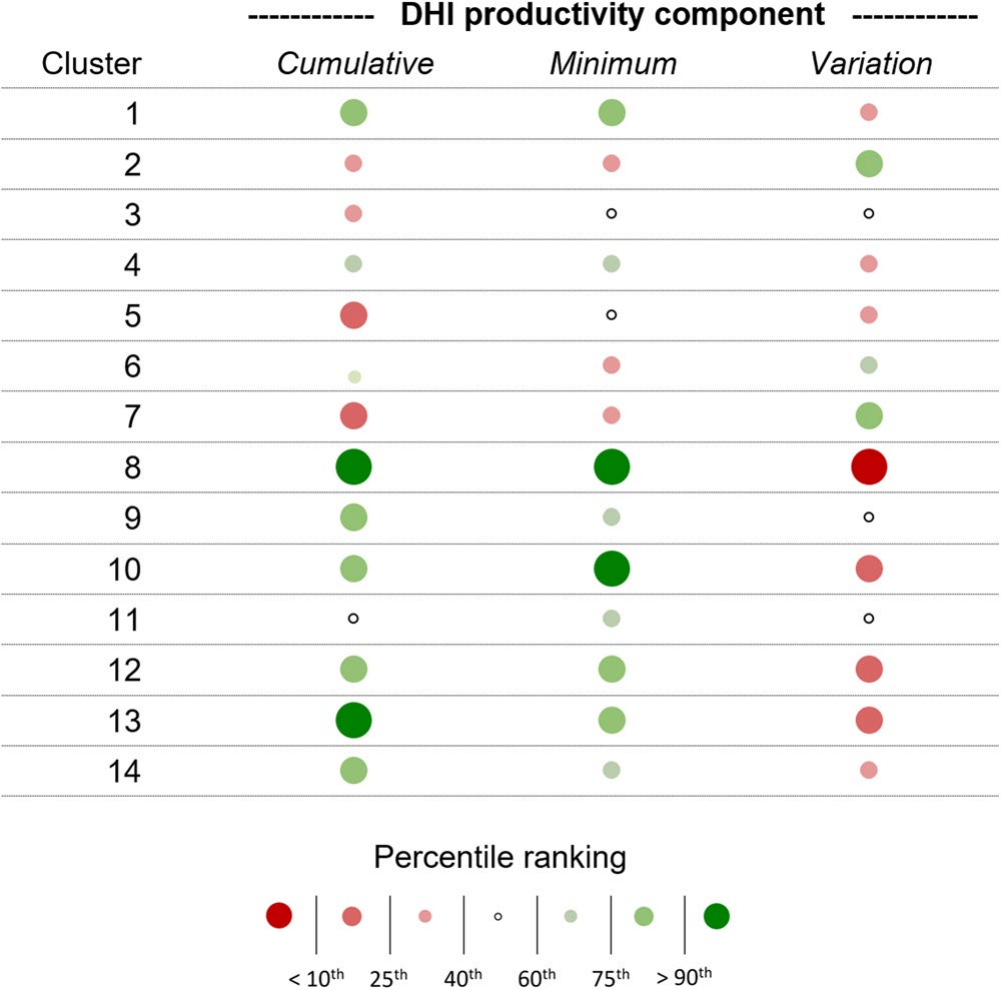
Relative percentile rankings by cluster for the three DHI productivity components. Percentiles are calculated from cluster mean values and circles represent one of seven discreet percentile ranges as indicated in the legend. Open circles indicate values between the 40^th^ and 60^th^ percentiles (i.e., relative median values). Below median values are colored red and above median values green. More extreme values are indicated by darker and larger circles.

At the 14-class level, the DHI-based clusters successfully identified unique regions of differing productivity and phenology characteristics over the planet, capturing major biomes such as the boreal, tropics, deserts and temperate regions (Fig. 2). Comparing the DHIs within each cluster, we found several distinct productivity groupings. Areas with very high productivity (cumulative and minimum) and very low seasonality (variation) included clusters 8 and 13 and, to a lesser extent, clusters 10 and 12 (Fig. 3). These clusters encompass the world’s tropical rainforests (clusters 8 and 13), such as the South American Amazon, the African Congo, much of Indonesia and elsewhere, as well as humid subtropical and coastal temperate forests (clusters 10 and 12). Mid-level productivity and low seasonality were typical for clusters 1, 4 and 14, including humid temperate and subtropical coniferous forests, and the moist savannas of South America, Africa, Asia and northern Australia. Mid-level productivity and moderate seasonality characterize clusters 6 and 9, which include the southern boreal (taiga) forests of North America, Russia and central Europe. Low cumulative productivity and moderate-to-high seasonality were typical for four clusters, spanning rainfed temperate croplands and grasslands (cluster 3; moderate seasonality), dry tropical scrublands and savannas (cluster 11; moderate seasonality), high-latitude steppes, shrublands and grasslands (cluster 2; high seasonality), and arctic shrublands and tundra (cluster 7; high seasonality). Low productivity and low seasonality were found in cluster 5, corresponding with arid and semi-arid vegetated regions, for example, in southern Africa, central Australia, and southwestern North America. As expected, given that the clusters were derived from the DHIs, the homogeneity and discriminant power of our regionalisation is high for each of the three DHIs, when compared to the 14 conventional global biomes developed by Olson *et al*.^6^ (Table 1).

**Table 1:** Within-region homogeneity of select variables.

Variable	— DHI clusters —			— Olson *et al*.^6^ biomes —		
	*CV*	*IQR-CV*	*L-CV*	*CV*	*IQR-CV*	*L-CV*
*DHIs*						
Cumulative fPAR				0.43	0.62	0.24
Minimum fPAR				1.58	0.99	0.47
Variation of fPAR				0.61	0.96	0.33
*Species richness*						
Amphibians	0.69	1.09	0.38	0.83	1.14	0.42
Birds	0.30	0.40	0.17	0.32	0.42	0.17
Mammals	0.36	0.47	0.20	0.39	0.57	0.22
Overall	0.30	0.41	0.17	0.32	0.43	0.17
*Other variables*						
Canopy height	0.92	0.77	0.46	1.14	0.84	0.56

Three measures of variation as indications of within-region homogeneity for the 14 Dynamic Habitat Index (DHI) clusters and the Olson *et al*.^6^ biomes: coefficient of variation (CV), interquartile range divided by the median (IQR-CV), and the second L-moment divided by the first L-moment (L-CV). Values are the mean value of each measure across all regions within each regionalisation, respectively. Note that individual DHI variables are not shown for the DHI clusters since they were used to derive the clusters.

In terms of spatial correspondence of the DHI clusters to the Olson *et al*.^6^ biomes, the greatest overlap occurred between the Tundra biome and cluster 7, the Deserts and Xeric Shrublands biome and cluster 5, and the Tropical Moist Broadleaf Forest biome and cluster 8 (Table 2). Other biomes tended to be split among several clusters, with the Temperate Broadleaf and Mixed Forest, the Temperate Coniferous Forest and the Flooded Grassland and Savannas biomes being the most diverse. Clusters 1 and 4 were among the largest clusters by area, and distributed across the most biomes, while clusters 13 and 14 were the smallest and concentrated primarily within a single biome, the Tropical Moist Broadleaf Forest.

**Table 2:** Spatial overlap between DHI derived clusters and Olson *et al*.^6^ global biomes.

*Total cluster area* (*million km*^*2*^)	DHI cluster														*Total biome area*
	*1*	*2*	*3*	*4*	*5*	*6*	*7*	*8*	*9*	*10*	*11*	*12*	*13*	*14*	
	9.80	13.51	10.81	14.02	13.55	5.43	9.38	10.54	3.07	3.53	5.31	3.73	2.73	0.51	
Percent area overlap with biomes*															
Tropical Moist Broadleaf Forest	13			14				47					13		19.51
	13			19				86		35		32	91	86	
Tropical Dry Broadleaf Forest			18	42								10			2.94
Tropical Coniferous Forest	26			29						19					0.69
Temperate Broadleaf and Mixed Forest	28	14	20			12			15						12.16
	34	12	23			26			57	19					
Temperate coniferous forest	24	17	15			14			11						3.91
	10					10			14						
Boreal Forest/Taiga		40				23	30								14.49
		42				60	46		24						
Tropical Grassland, Savannas, Shrubland				43	11						21	11			18.72
	11			57	14					20	72	52			
Temperate Grassland, Savannas, Shrubland	12	40	29		15										9.77
	12	28	26		11										
Flooded Grassland and Savannas	10	16		23						11	19				0.92
Montane Grassland and Shrubland			32		35										3.80
			11												
Tundra							90								5.84
							52								
Mediterranean Forests, Woodlands and Scrub	40		23		29										2.79
	11														
Deserts and Xeric Shrubland			17		61										12.63
			18		54										
Mangroves				23				30				12	11		0.29

*Upper left value is the precent area of each Olson *et al*.^6^ biome by DHI cluster; lower right value is the percent area of each cluster by biome. Values are only shown when ≥10% and therefore rows (upper left) and columns (lower right) may not sum to 100%.

**Figure 4:**
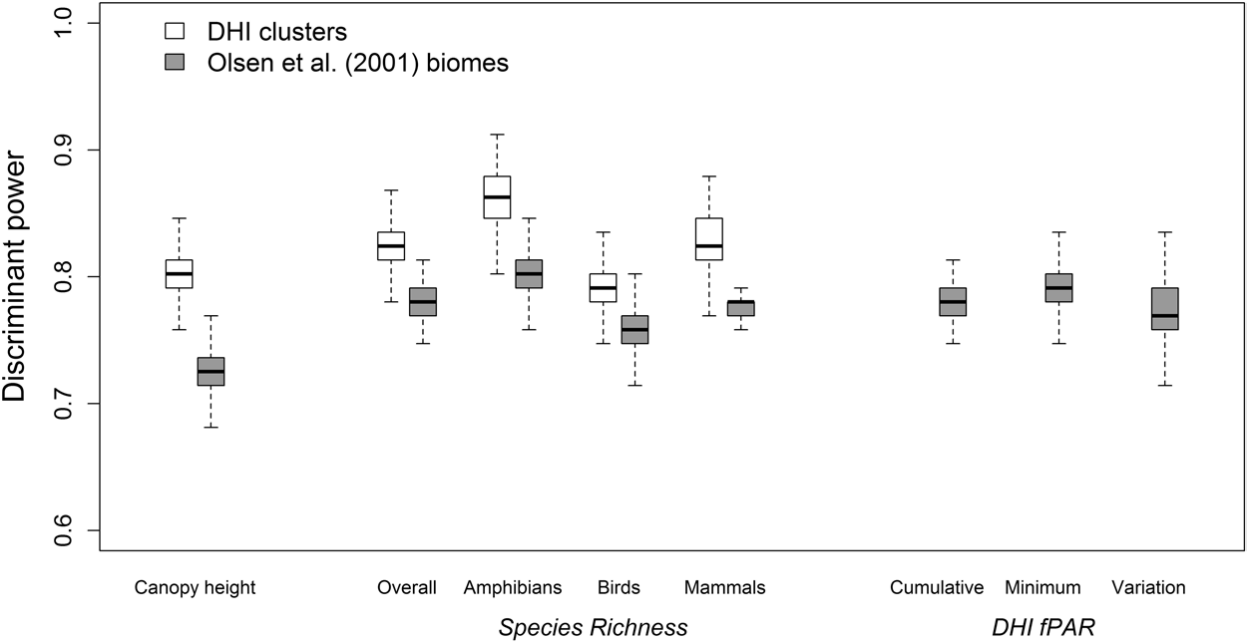
Discriminant power of DHI clusters and Olson *et al*.^6^ biomes. Discriminant power is the proportion of all possible Games-Howell pairwise rank comparisons that are significant at p < 0.05. Pairwise comparisons were repeated 500 times using random samples (n = 100) drawn from each region within the DHI clusters and Olson *et al*.^6^ biomes, respectively. Horizontal lines represent median discriminant power values, boxes are the interquartile range (IQR; 25^th^ and 75^th^ percentiles) and whiskers are the smallest and largest values within 1.5*IQR of the 25^th^ and 75^th^ percentiles, respectively. Note that individual DHI variables are not shown for the DHI clusters since they were used to derive the clusters.

Within-region variation of species richness and canopy height of both the DHI clusters and the Olson *et al*.^6^ biomes confirmed that across regions, the DHI clusters were more homogenous for all variables evaluated (Table 1). Between-region discriminant power was also higher for the DHI clusters (Fig. 4). For overall species richness, the DHI clusters were 6% more likely to have significant differences between clusters than the Olson *et al*.^6^ biomes. For amphibians, birds and mammals, increases in discriminant power for the DHI clusters were 8%, 4% and 6%, respectively. Comparing the DHI-based clusters to global tree canopy height showed also that the DHI clusters distinguished height classes better than the Olson *et al*.^6^ biomes.

**Figure 5:**
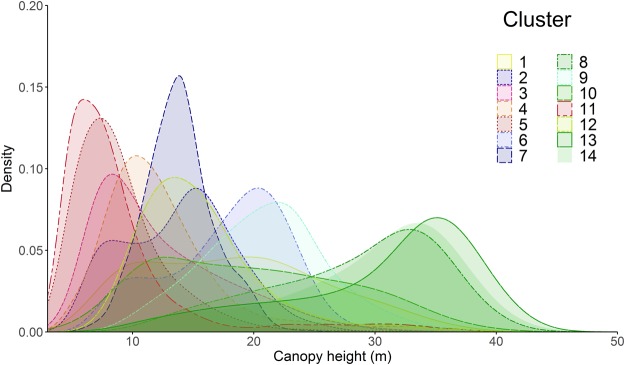
Density plot of canopy height for each DHI-derived cluster. Smoothed Gaussian kernel density plot of the distributions of canopy height (>0 m) based on a stratified random sample from each cluster. Green colors indicate higher productivity (cumulative fPAR), blue colors indicate higher seasonality (variation of fPAR) and red colors indicate low productivity and low seasonality. See Fig. 2 for a legend and more detailed description of cluster color assignments.

Examining the distribution of tree heights by DHI clusters (Fig. 5) showed broad trends, with the tallest trees (>30 m) occurring in clusters with high cumulative productivity and low seasonality (clusters 8, 13 and 14; i.e., tropical rainforests). These clusters also have relatively wide distributions of their canopy heights, suggesting they are mature forests with complex multi-strata canopy structures. The clusters with moderately high productivity and seasonality (clusters 6 and 9) exhibit medium tree heights from 15–25 m, indicative of the intact southern boreal forests. The shortest trees are associated with clusters with moderate-to-low productivity and low seasonality (e.g., clusters in arctic shrublands and tundra, high-latitude grasslands and semi-arid savannas and shrublands) and distributions generally became narrower as mean canopy height decreases. Two clusters (1 and 10) with high productivity and low seasonality had relatively uniform canopy height distributions and variable tree heights. Incidentally, these clusters corresponded to areas with intensive human management, which may be altering relationships between DHI and canopy height, for example by reducing canopy heights (e.g., forest clearing) or increasing productivity (e.g., irrigated agriculture).

**Figure 6:**
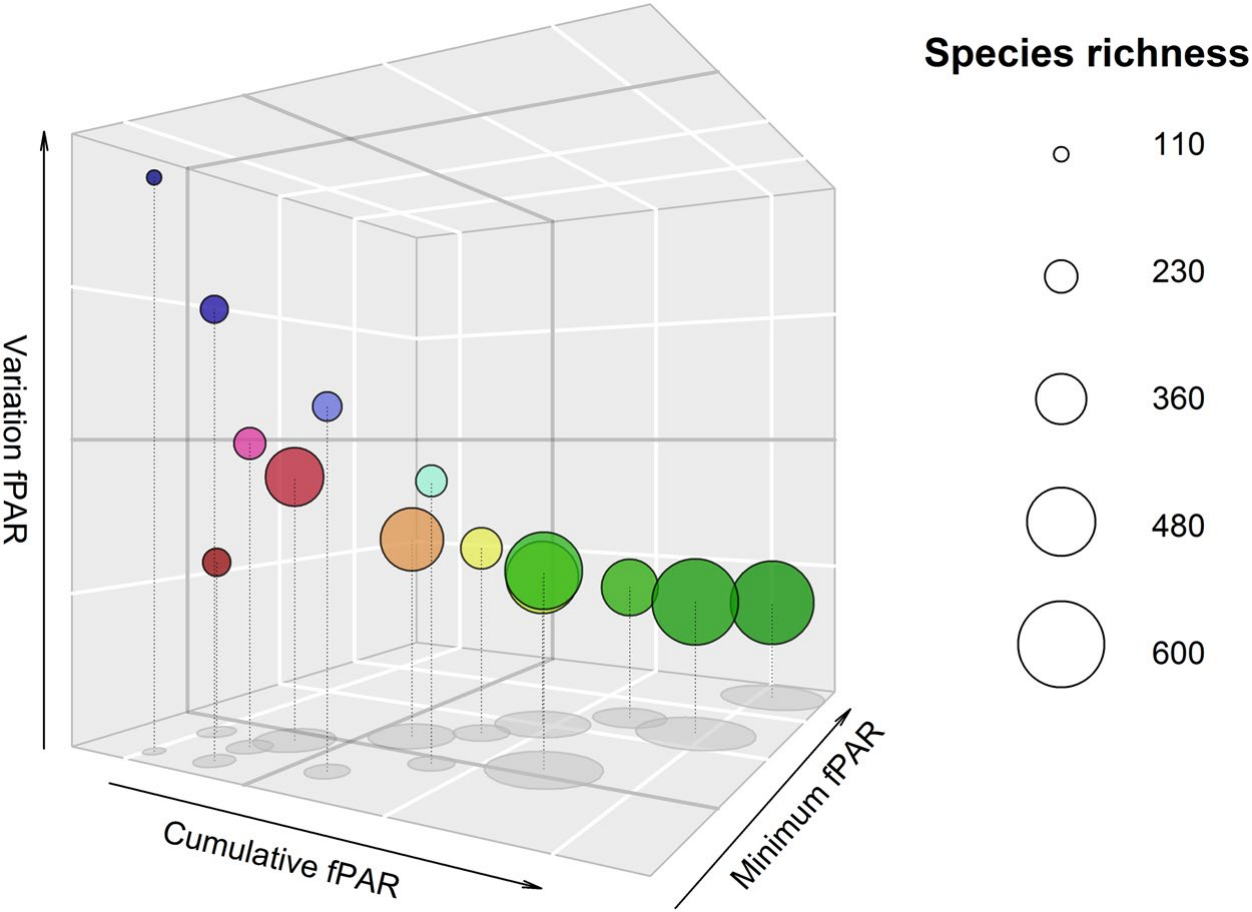
Three-dimensional scatter plot of z-scores of DHI input variables for 14 clusters. Axes are the z-score of each variable, with z-score = 0 in dark grey and each white tick representing a z-score of 1. Point centers represents the mean z-score value of each of the 14 DHI derived clusters. Point size reflects overall combined species richness of birds, mammals and amphibians. Green clusters indicate higher productivity and lower seasonality (e.g., rainforests), blue areas indicate higher seasonality and lower productivity (e.g., boreal forests), and red areas indicate low productivity and low seasonality (e.g., deserts). See Fig. 2 for a legend and more detailed description of cluster color assignments.

Comparing species richness across the three DHIs, as grouped by the 14 DHI clusters, highlighted that as cumulative productivity increases, minimum productivity tended to increase and seasonality (productivity variation) decreased (Fig. 6), as ecological theory would predict. Where cumulative productivity of DHI is very low, species richness is low irrespective of whether seasonality was low (deserts) or high (arctic and boreal). At intermediate levels of cumulative productivity, species richness tended to be higher as seasonality decreased and minimum productivity increased.

**Figure 7:**
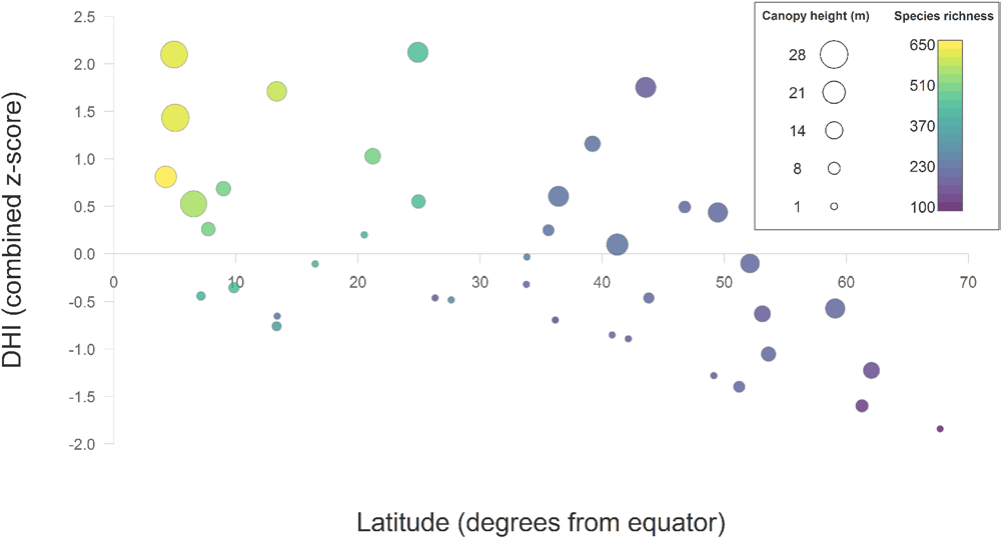
Scatter plot showing the relationship between DHI, latitude, canopy height and species richness for 40 clusters. Points represent the mean values for each of each of the 40 DHI-derived clusters. The DHI combined z-score was calculated as the sum of scaled cumulative and minimum fPAR and the scaled inverse of variation of fPAR (seasonality). Points are sized by their mean canopy height (in meters) and colored by mean overall species richness of birds, mammals, and amphibians together.

Cluster validity metrics stabilized at around 40 clusters, suggesting that this may be the maximum number of meaningful DHI-derived clusters. Increasing the number of DHI clusters from 14 to 40 increased the mean homogeneity of the DHI clusters for most variables (data not shown), and affected the relationship between DHI components, latitude, canopy height and species richness somewhat (Fig. 7). Broad global trends in species richness were strongly related to latitude, with the greatest values observed near the equator. More regional trends (i.e., for a given latitude) were related to the three DHIs and canopy height: both species richness and canopy height were greater with increasing DHI combined z-scores (higher cumulative and minimum productivity and lower seasonality). This relationship was especially pronounced at latitudes within about 30 degrees of the equator.

## Discussion

The DHIs reflect a number of environmental parameters, including climate and terrain, as well as information of vegetation production, and in some instances land cover and land use patterns, and that makes them powerful predictors of biodiversity patterns^35^. Conventionally, these factors have been individually related to species occurrence or abundance^36^. However, because the DHIs are computed based on 8-day variations in fPAR, which is a key productivity indicator, they provide a link with previous experimental, descriptive, and theoretical work that relates productivity to species richness and composition^37,38^.

We choose fPAR as the metrics upon which to derive DHIs for this work, as opposed to other satellite measures of landscape greenness, such as NDVI or EVI, for several reasons. MODIS predictions of fPAR are derived from physically based models of the propagation of light in plant canopies^39,40^. As a result, the MODIS fPAR model utilizes more than two spectral bands (up to 7), not just the red and near-infrared reflectance, as the NDVI does, or red, near-infrared and blue reflectance, as in the case of the EVI. Furthermore, the fPAR retrieval considers sun angle, background reflectance, and view angle influences, which simple vegetation ratios do not. However, fPAR estimates can be noisy due to snow, cloud, and low sun angle^41^. The global analysis of the DHIs^28^ is thus based on a single composite phenology curve from all MODIS fPAR data from 2003–2014 rather than from a single year, greatly reducing noise in the derived DHIs.

The DHI derived clusters based on variations in fPAR throughout the year better discriminated the variation in species richness for three taxa globally than existing global regionalisation maps. This result very much surprised us, because the biome map to which we compared our cluster was based on species data. Furthermore, the DHI-based clusters captured global variability of a key habitat variable in forested ecosystems, i.e., tree height, better than the conventional biome map. Given rapid global change and threats to biodiversity and ecosystems, and the need to make conservation more efficient, ecological regionalisation maps have become a vital dataset for conservation planning, and recent work has emphasized the potential to use remotely sensed data to quantitatively map ecoregions and enable timely, accurate and statistically sound regionalisations^11,42–45^. However, so far most quantitative regionalisation approaches have limited their data inputs to environmental variables representing climate, topography, and edaphic factors^11,17,42,43,45–49^. The use of remotely sensed data as the basis for regionalisations has a number of key benefits over both conventional approaches and climate data. First and foremost, regionalisations based on satellite data are more amendable to change, because new remotely sensed data can be readily added to allow the regionalisation to adapt to changes in terrestrial conditions either due to climate or land use change. Second, regionalisation based on remotely-sensed data can capture fine-scale spatial patterns within what are traditionally large, contiguous ecoregions (Fig. 2, insets a, b and c). As discussed by Olson *et al*.^6^ one caveat to conventional ecoregions is that some regions may contain habitats that differ from their assigned biome, for example open wetlands in boreal forest, or savannas in the rainforests of Amazonia. By identifying clusters at the 1-km scale, such patterns can easily be regionalised at multiple scales. Lastly, climate data available as interpolated surfaces rely on a network of whether stations and do not provide actual gridded measurements as remotely sensed data do.

Having said that, regionalisations based on remotely sensed data also have inherent disadvantages.

In this paper we utilized a clustering based approach rather than a segmentation of the DHI layers. Using our approach, the DHI pixels are agglomerated into larger zones (clusters) using the scaled Euclidean distance in dataspace. As is evident in the results, a clustering based approach does not require clusters to be continuous and as a result cells with similar DHI attributes can be assigned to the same cluster even if they are geographically distinct. This can lead to fragmented clusters which is not an issue for understanding relationships between environment and species for example, but may make the approach less well suited to mapping^50^. A segmentation-based approach provides an alterative which not only develops clusters based on their environmental similarity but also their spatial relationships, producing spatially contiguous clusters which may be more useful for mapping. This approach, while attractive for map makers, is computationally very expensive, and often is undertaken at broader spatial scales than the 1 km analysis in this research (i.e., 30 km^50^) and will invariably involve grouping cells which are in fact environmentally distinct in the same cluster simply due to location.

By restricting ourselves to remotely-sensed data, our regionalisations did not account for evolutionary history, patterns of endemic genera and families, distinct assemblages of species, and geological history (e.g., glaciations or Pleistocene land bridges), and their effects on the distribution of plants and animals^6^. Furthermore, a productivity-driven regionalisation encapsulates a variety of ecosystem processes such as climate, photosynthesis, phenology, vegetation age and disturbance in a single metric, in our case fPAR, making if difficult to disentangle which of these processes are the critical ones driving ecosystem regionalisation. This is also the case with land use, which can alter the productivity of the landscape through fertilisation or other management activities. As a result, patterns in regionalisations may be governed, at a landscape level, by factors beyond variations in natural vegetation. Despite these caveats though, our results highlight the promise of using remotely-sensed data to make regionalisations even more valuable for ecosystem management and conservation.

## Methods

### Dynamic Habitat Indices

The concept of the Dynamic Habitat Indices (DHIs) were originally developed by Mackey *et al*.^26^ and Berry *et al*.^27^ in Australia and updated by Coops *et al*.^19^ in North America, and globally by Hobi *et al*.^51^ and Radeloff *et al*.^28^. The DHIs provide three dimensions of biodiversity through three individual indices that can be combined visually or statistically. The three indices include annual integrated measures of (a) the cumulative annual productivity as the integrated landscape productive capacity over a year, (b) the annual minimum productivity as the minimum amount of vegetation production over a year, and (c) annual seasonal variation in productivity which reflects how the vegetation varies within the year, an indicator of climatic variation, and phenology. The DHI can be computed from a temporal sequence of remote sensing observations including, the Normalized Difference Vegetation Index (NDVI), leaf area index (LAI), the fraction of light absorbed by the vegetation (fPAR), or estimates of Gross Primary Productivity (GPP)^51^. Irrespective of the type of productivity measure, it is necessary to summarize the satellite observations throughout the course of the year in order to evaluate the DHI.

Previous research into the application of the DHIs as possible indicators of aspects of biodiversity have shown that they correlate well with species richness conducted at landscape and continental scales. Coops *et al*.^52^ and Hobi *et al*.^51^ both found cumulative DHI to be significantly correlated with avian species richness across the United States when compared to breeding bird survey data. In Canada, grassland bird species richness was highly correlated with both the minimum DHI and annual variation in DHI^52^. Additional research has been undertaken examining DHIs and beta diversity of butterfly communities (which was positively correlated with minimum DHI and cumulative DHI^53^ and moose (*Alces americanus*) occurrence and abundance models^54^. The DHIs have also been used regionally to drive ecoregion mapping for the boreal forests of Canada^55^.

Processing of the global DHIs is described in Radeloff *et al*.^28^ and therefore only briefly detailed here. We utilised Eight-day MODIS fPAR layers to be consistent with most DHI studies thus far^19,53,54^ and downloaded data from the MODIS DAAC with GeoTIFFS then derived from the HDF files. Individual tiles were mosaicked to produce global datasets for each time step. Only high quality screen pixels (quality assessment <83) were considered in the analysis and all land cover types were processed (except deserts, snow and ice), over all terrestrial land globally except Antarctica, and islands. All DHIs are available for download at http://silvis.forest.wisc.edu/data/DHIs. The calculation of DHIs can be sensitive to noise, which is why we analyzed a single composite phenology curve from all MODIS data from 2003 to 2014 rather than single-year DHIs. The composite phenology curve represents the median value for each of the 12 observations that were available for each of the 46 time steps available in the 8-day MODIS fPAR product.

### Canopy height

While estimates of leaf area and fPAR based on optical remote sensing data have been shown to be sensitive and dynamic indicators of overall vegetation productivity^56^, optical remote sensing is not well suited for capturing the vertical structure of vegetation^57^. Alternatively, Light Detection and Ranging (LiDAR), an active form of remote sensing, can directly measure the vertical structure of vegetation^58,59^.

In order to determine if DHIs captured variability in canopy height in this study, we obtained a LiDAR-based global canopy height product developed by Simard *et al*.^34^. Simard *et al*.^34^ utilized a global sample of LiDAR data collected by the Geoscience Laser Altimeter System (GLAS) aboard the Ice, Cloud and land Elevation Satellite (ICESat), which collected waveform LiDAR data globally from 2003 to 2009. GLAS laser footprints are ~65 m in diameter and separated by 172 m along track and up to 14.5 km across tracks^60^, providing a sample of forest structure over the globe. Simard *et al*.^34^ derived global wall-to-wall estimates of canopy height by extrapolating GLAS derived canopy heights through empirical relationships with spatially contiguous variables^34,61^. Specifically, Simard *et al*.^34^ used cloud-free GLAS waveforms acquired from May and June of 2005 (L3C) and climate, topography, and other globally available ancillary variables to predict canopy height for each GLAS waveform modified by a slope map using 90-m Shuttle Radar Topography Mission (SRTM) data to correct potential bias. Simard *et al*.^34^ removed all waveforms from the analysis that were located in areas of high slope (>5 degrees) or where the slope correction was >25% of the measured waveform and applied a Random Forest model to extrapolate values based on seven globally available variables: mean precipitation, precipitation seasonality, mean temperature, temperature seasonality, elevation, MODIS tree cover, and protection status.

### Species Richness Data

Global layers of species distributions are available through the International Union for the Conservation of Nature (IUCN), and BirdLife International covering range maps for amphibians, bird, and mammals^62,63^. These layers have formed the basis of previous global biodiversity studies^64–67^. All range maps were converted to rasters with 1-km resolution, matching the native resolution of the MODIS DHIs datasets, and we produced global species richness maps for amphibians, birds, and mammals by counting a species as present if any part of the grid cell was within the species’ range polygon.

### Conventional regionalisations

The Terrestrial Ecoregions of the World (TEOW), derived by the World Wildlife Fund (WWF), provides a widely-applied example of a global biogeographic regionalisation^6^. The TEOW regionalisation defines relatively large units of land containing distinct assemblages of natural communities sharing a large majority of species, dynamics, and environmental conditions. In total 867 terrestrial ecoregions, classified into 14 different biomes, were defined. The regionalisation is similar to other subjectively derived regionalisations in that it brings together a suite of existing biogeographic maps developed by others^68–71^ and compares them with global and regional distributions of plants, and animals^6^. The biomes and terrestrial ecoregions are available for download at https://www.worldwildlife.org/publications/terrestrial-ecoregions-of-the-world.

### Clustering

In order to identify unique regions based on the DHIs, we implemented an unsupervised (i.e., unconstrained) two-stage clustering approach similar to that proposed by Tamura *et al*.^72^, which has previously been used for ecological regionalisation at sub-continental scales^17,45,73,74^. The two-stage approach combines the computational speed of k-means clustering (stage one) with agglomerative hierarchal clustering (stage two), which recursively merges the initial k-mean clusters, but on its own is not suitable for very large datasets due to computational constraints. The two-stage process therefore allows for the processing of a large number of pixels and input variables via k-means, while retaining the benefits of hierarchical clustering, such as the nested structure and the flexibility to determine the final number of clusters using validity metrics.

In the first step, we generated 867 pre-clusters using a one-pass ‘k-means++’ algorithm, equal to the number of terrestrial ecoregions developed by Olson *et al*.^6^. In the second step, we combined pre-clusters based on the similarity of their centroids in the three-dimensional feature space defined by the DHIs using agglomerative hierarchical clustering. We masked pixels with a cumulative fPAR equal to zero from analysis, removed outliers, and rescaled all input data prior to the initial k-means pre-clustering. Latitude (in degrees from the equator) was also included as an input variable to account for global-scale temperature gradients that occur across similar productivity levels, and to reduce the likelihood of disparate individual pixels being assigned to a cluster geographically distant. Hierarchical clustering was performed using the ‘Ward’ linkage algorithm^75,76^ and Euclidian distances between pre-cluster centroids. The resulting dendrogram was first cut at 14 clusters – equal to the number of global biomes developed by Olson *et al*.^6^ – using dynamic tree cutting to prune branches based on their structure in the dendogram, which can improve automation with complex dendrograms compared to using a static cut-off at a fixed height^77^. The dendrogram was also cut at 40 clusters, which we estimated to be the maximum number of meaningful clusters based on a visual assessment of multiple cluster validity metrics (e.g., Silhouette score, between- and within-cluster separation, within-cluster sum of squares).

All clustering outputs were ‘sieved’ to achieve a minimum map unit size of 20 km^2^. This process does not change the final resolution of the map, but rather iteratively removes isolated regions with fewer than 20 contiguous pixels and assigns them the value of the majority nearest neighbors.

### Statistical analyses

We evaluated the variance of eight variables related to the DHIs, biodiversity, and canopy height (Table 1) within and among the 14 DHI-derived clusters and compared it to the 14 biomes developed by Olson *et al*.^6^. Variation among biomes was assessed using measures of within-region homogeneity and an estimation of between-region discrimination. Within-region homogeneity was quantified as the mean coefficient of variation across all regions within each of the two regionalisations, respectively. The coefficient of variation was calculated using three different methods to account for differing data distributions: the mean divided by the standard deviation (CV), the interquartile range divided by the median (IQR-CV), and the ratio of the second and first L-moments (L-CV), a statistic used in hydrological regional frequency analysis^78^. Spatial overlap of the DHI clusters and the Olson *et al*.^6^ biomes was calculated in ArcGIS 10.5 (http://www.esri.com/software/arcgis/arcgis-for-desktop) to assess the spatial correspondence between the two regionalisations. While the results of this method are difficult to interpret (Table 2), other methods to quantify spatial associations between regionlisations such as the informational-theoretical V-measure^79^ and Mapcurves^80^ were attempted and deemed computationally infeasible given the global scale and fine spatial resolution of the DHI clustering.

Between-region discrimination was assessed using a measure of the ‘discriminant power’ for each regionalization (Fig. 4). We defined discriminant power as the likelihood that, when sampled, two regions within a given regionalisation approach will have significantly different means for a given variable. We estimated discriminant power by first drawing a random sample (n = 100) of each variable from each region and performing all possible pairwise comparisons between all region pairs within a given regionalisation. To control for type-1 error and allow for non-constant variance, pairwise comparisons were made using nonparametric Games-Howell tests^81^. Discriminant power was defined as the number of significant pairwise differences (at p < 0.05) divided by the total number of pairwise comparisons, yielding a value ranging from 0 to 1. We repeated the sampling and pairwise comparison process 500 times to produce a distribution of discriminant power values of each variable for the DHI clusters and the Olson *et al*.^6^ biomes, respectively.

In addition, we visually assessed density and scatter plots to identify differences among DHI clusters and examine relationships between the DHIs, species richness and canopy height. Statistical analysis and plotting was performed in R v3.4.1^82^. Data pre-processing and k-means pre-clustering were performed using *ArcPy*^83^ and the *scikit-learn* package^84^ in Python v2.7. The agglomerative hierarchical clustering step was performed in R using the *stats* package^82^ to produce the complete dendrogram and the *dynamicTreeCut* package^77^ for dynamic branch pruning.

## Data Availability

All datasets are freely available, no restrictions are placed on their use, and they can be shared and redistributed. For questions, please contact Volker Radeloff (radeloff@wisc.edu), and we love to learn about applications of the DHIs, and appreciate notifications when there are problems with the datasets. When referring to the DHIs please cite: Hobi, M.L., Dubinin, M., Graham, C.H., Coops, N.C., Clayton, M.K., Pidgeon, A.M. & Radeloff, V.C. (2017). A comparison of Dynamic Habitat Indices derived from different MODIS products as predictors of avian species richness. Remote Sensing of Environment, 195, 142–152. The derived species richness surfaces and the 3 levels of clusters are also available for free download from http://silvis.forest.wisc.edu/data/DHIs-clusters.
